# Supplementary material for: An interpretable deep learning framework for predictive modeling of postoperative infections in ICU patients
Source: PLoS One. 2026 Apr 9;21(4):e0346896. doi: 10.1371/journal.pone.0346896 (PMC13065004; doi:10.1371/journal.pone.0346896)
Supplement: Attachment — Submitted filename: Response.pdf [file pone.0346896.s002.pdf]

Responses to Journal Requirements

1. Please ensure that your manuscript meets PLOS ONE's style requirements, including those for file naming.

Response: Confirmed.

2. We noticed you have some minor occurrence of overlapping text with the following previous publication(s), which needs to be addressed: Precision Opioid Prescription in ICU Surgery: Insights from an Interpretable Deep Learning Framework - https://doi.org/10.29011/2575-9760.11189 In your revision ensure you cite all your sources (including your own works), and quote or rephrase any duplicated text outside the methods section. Further consideration is dependent on these concerns being addressed.

Response: Thanks for catching this. It has been addressed.

3. Thank you for stating in your Funding Statement: "This study was partially supported by NIH (National Institutes of Health) R56 (1R56LM013784) and R01 (R01LM014407 and 1R01HL173044) grants."

*Please provide an amended statement that declares *all* the funding or sources of support (whether external or internal to your organization) received during this study, as detailed online in our guide for authors at http://journals.plos.org/plosone/s/submit-now.*

Please also include the statement "There was no additional external funding received for this study." in your updated Funding Statement. Please include your amended Funding Statement within your cover letter. We will change the online submission form on your behalf.

Response: Done.

4. Please state what role the funders took in the study. If the funders had no role, please state: "The funders had no role in study design, data collection and analysis, decision to publish, or preparation of the manuscript."

If this statement is not correct you must amend it as needed. Please include this amended Role of Funder statement in your cover letter; we will change the online submission form on your behalf.

Response: Funding Statement has been revised as "This study was partially supported by NIH (National Institutes of Health) R56 (1R56LM013784) and R01 (R01LM014407 and 1R01HL173044) grants. There was no additional external funding received for this study. The funders had no role in study design, data collection and analysis, decision to publish, or preparation of the manuscript."

5. We note that you have provided funding information that is currently declared in your Funding Statement. However, funding information should not appear in the Acknowledgments section or other

areas of your manuscript. We will only publish funding information present in the Funding Statement section of the online submission form.

Please remove any funding-related text from the manuscript and let us know how you would like to update your Funding Statement. Please include your amended statements within your cover letter; we will change the online submission form on your behalf.

Response: Removed.

6. We note that you have indicated that there are restrictions to data sharing for this study. For studies involving human research participant data or other sensitive data, we encourage authors to share de-identified or anonymized data. However, when data cannot be publicly shared for ethical reasons, we allow authors to make their data sets available upon request.

Response: Though the data is publicly available, it can not be shared publicly since the access to the data should be granted by the data owner. Instead, a Python script used to extract the data along with the analytic codes are provided and specified in Data Availability section.

Before we proceed with your manuscript, please address the following prompts:

a) If there are ethical or legal restrictions on sharing a de-identified data set, please explain them in detail (e.g., data contain potentially identifying or sensitive patient information, data are owned by a third-party organization, etc.) and who has imposed them (e.g., a Research Ethics Committee or Institutional Review Board, etc.). Please also provide contact information for a data access committee, ethics committee, or other institutional body to which data requests may be sent.

b) If there are no restrictions, please upload the minimal anonymized data set necessary to replicate your study findings to a stable, public repository and provide us with the relevant URLs, DOIs, or accession numbers. Please see http://www.bmj.com/content/340/bmj.c181.long for guidelines on how to de-identify and prepare clinical data for publication. For a list of recommended repositories, please see https://journals.plos.org/plosone/s/recommended-repositories. You also have the option of uploading the data as Supporting Information files, but we would recommend depositing data directly to a data repository if possible. Please update your Data Availability statement in the submission form accordingly.

Response: Done.

Responses to comments from Reviewer 1

1) Some terminology is introduced without precise definition (e.g., "stable DNN model") or where claims are made without supporting citations (e.g., about the limitations of interpretability in existing ML models used in ICU prediction).

However, the current version of the manuscript overstates the practical utility of the approach given the results.

While the DNN ensemble performs marginally better than existing models, the improvement in AUC and PR-AUC is incremental, and the low sensitivity across all methods suggests that the model may not be suitable for critical clinical use without further refinement. The lack of external validation, absence of confidence intervals or statistical comparisons of feature importance, and limited discussion of generalisability or deployment constraints also weaken the broader applicability of the findings.

More thorough statistical evaluation of model outputs, particularly in terms of calibration and variance.

Response: Thanks for the comments, suggestions and sorry for the confusions. As we have emphasized in the Introduction section that the focus of this paper is on how to robustly identify critical features associated with postoperative infection under potential complex association relationship, which is not available in existing research. Specifically, existing methods for identifying important features associated with postoperative infections are parametric methods requiring specifying the functional relationship expressively (i.e., linear additive assumption). However, this assumption can not be verified and hardly hold in practice. To address this difficulty, we 1) adopt the stable deep neural network model for robustly modeling the potential complex association between clinical features and postoperative infection risk; 2) apply the permutation feature importance test to evaluate the impact of each input feature associated with postoperative infection risk when a black-box (machine learning) model is adopted such that the impact of each input feature can be evaluated rigorously which is clinically important in practice. We totally agree that the prediction performance based on DNN is marginally better than other competing methods. However, most of the important features identified by DNN have been well established in previous clinical studies while other competing methods frequently failed to capture these features which raised the concern of the reliability for these methods to identify the important factors associated with postoperative infection. We have added more comments in the Introduction, Results, and Discussion section in the revised paper.

2) Additional detail on how the DNN ensemble was constructed, filtered, and how unstable models were defined or excluded.

Response: More details have been added in the revised paper in Methods section.

3) Clarification of feature selection strategy and justification for excluded clinically relevant features such as smoking status or nutritional indicators.

Response: We completely agree that these are important factors that should be included. However, they

are not available in this data set.

4) Deeper comparison of the PermFIT approach to existing interpretable ML methods (e.g., SHAP, LIME).

Response: Thanks for the information provided. Indeed, in our paper (“Permutation-based Identification of Important Biomarkers for Complex Diseases via Machine Learning Models". *Nature Communications*, 12(1):3008), we have conducted comprehensive comparisons with many other feature importance tools including SHAP and LIME. Our PermFIT method proposed in the aforementioned paper outperformed all methods considered. Also, it should be noted that SHAP and LIME don’t offer rigorous statistical inference when evaluating a feature’s impact while the proposed PermFIT method not only provided principled statistical inference but also the relative feature importance ranking. As such we adopted PermFIT along with the stable DNN to identify important features associated with postoperative infection. We have made more comments in the revised paper in Introduction section.

Responses to comments from Reviewer 2

Reviewer #2: The study is well-motivated and relevant to PLOS ONE's scope, but several critical issues require clarification or improvement before the manuscript can be considered for publication. Major Scientific Comments and Questions

Minor Issues and Suggestions

• Clarify acronym definitions at first use (e.g., EHR, DNN).

• Move all figures and tables closer to their first mention for better readability.

• Ensure all references (e.g., Ref [39] for MIMIC-III) link to publicly accessible data sources, and include IRB or ethical approval statements if applicable.

• Typo: "healthcare-associated infections" is occasionally misspelled as "healthcare-associate infections."

1) While the PermFIT approach is emphasized as novel, the authors cite their own previous work (Mi et al., 2021; Ref [41]) that also introduced a similar DNN model. How does this work advance that prior publication?

Response: Thank you for the comments and valuable suggestions. Yes, you are correct that both PermFIT and DNN model adopted in this paper are from our earlier studies. The motivation of this paper is to introduce a robust and powerful framework proposed in our earlier studies to identify important features associated with postoperative infections which is not available in existing postoperative infection research. As we have clarified in the Introduction section that existing methods for identifying important features of postoperative infection are all parametric or semi-parametric based methods where strong assumption, i.e., generalized linear additive assumption is made. However, this assumption can not be verified and barely hold in practice. To address this critical difficulty, we adopt our proposed PermFIT and DNN methods to identify important features for postoperative infection under potential complex association without the need to make strong generalized linear additive assumption. We have added more comments in the revised paper.

2) Despite high AUC and specificity, sensitivity is very low (0.32), meaning a large proportion of infected patients would be missed. Is this acceptable for a clinical prediction model?

Response: We totally agree that sensitivity is very low potentially due to the fact that some other important features may not have been included in the model due to their unavailability. However, as we indicate in Introduction section, the motivation of this paper is to introduce a robust and powerful framework to identify critical features associated with postoperative infection under potential complex association setting. Also, most of the important features identified by our DNN model have been clinically verified. On the other hand, other competing methods failed to identify some of these clinically verified important

features. This clearly suggests that with more data and more comprehensive features being collected our DNN model could capture more comprehensive critical features for postoperative infection and improve the risk prediction accuracy and enhance postoperative pain management. More comments have been added in Results and Discussion section in the revised paper.

3) The authors mention 25 input features but do not explain why these were chosen or whether other known infection risk factors (e.g., surgical duration, antibiotic use) were considered.

Response: Thank you very much for the information. We totally agree that surgical duration and antibiotic use are important factors associated with postoperative infection risk. However, they are not available in the data set we analyzed. We chose baseline demographic, preoperative, and operative features to be used to predict the postoperative outcome, i.e., postoperative infection and identified the associated important features. There are total 25 available baseline demographic, preoperative, and operative features in this data set. More details have been added in the revised paper.

4) How were categorical variables encoded? Were patients with multiple ICU admissions properly deduplicated? Could leakage from label-informed preprocessing have inflated results?

Response: As usual, we created dummy variables to encode categorical variables such as race, gender, etc. Specifically, for a q category variable, we created q-1 dummy variables. Also, there is no leakage issue since all the evaluations were based on testing samples where their information was not used to train the model.

5) The study uses MIMIC-III exclusively. Have the authors tested the model on MIMIC-IV or another external dataset?

Response: We did not test the model on MIMIC-IV or other external dataset since most of the important features identified by the introduced DNN method were clinically verified in previous studies while other competing methods failed to capture many of these features. Also, this is the largest publicly available ICU cohorts. We have included more details in the Results section in the revised paper.

6) How were p-values corrected for multiple hypothesis testing (e.g., Bonferroni or FDR)? With 25 features, there's a high risk of Type I error.

Response: We totally agree that the multiple comparisons should be adjusted for the identified features to be claimed as statistically significant. However, the focus of this paper is on how to robustly identify critical features associated with postoperative infection under the complex association relationship, which is not available in existing research. To address this critical challenge, we 1) adopt the stable deep neural network model for robustly modeling the potential complex association between clinical features and postoperative infection risk; 2) apply the permutation feature importance test framework to evaluate

the impact of each input feature associated with postoperative infection risk when a black-box model is adopted such that the impact of each input feature can be evaluated rigorously which is clinically important in practice in postoperative pain management. We have added more comments in the Introduction section.

7) The authors report high specificity but low sensitivity. Were additional metrics such as F1-score, Brier score, or calibration curves considered?

Response: Thank you for the suggestion. F1-score is reported in Table 1 in the revised paper. Please note that the focus of this paper is to introduce a robust and powerful framework to identify important features associated with postoperative infection under potential complex association settings. Furthermore, we have reported cut-off dependent metrics (e.g., Accuracy, Specificity, and Sensitivity) and cut-off independent metric (e.g., AUC, PR-AUC) and these are commonly used for evaluating a classification model's performance where F1-score is also a cut-off dependent metric.
