# Supplementary material for: An interpretable deep learning framework for predictive modeling of postoperative infections in ICU patients
Source: PLoS One. 2026 Apr 9;21(4):e0346896. doi: 10.1371/journal.pone.0346896 (PMC13065004; doi:10.1371/journal.pone.0346896)
Supplement: Attachment — Submitted filename: Response to Reviewers.pdf [file pone.0346896.s003.pdf]

Responses to Journal Requirements

1. If the reviewer comments include a recommendation to cite specific previously published works, please review and evaluate these publications to determine whether they are relevant and should be cited. There is no requirement to cite these works unless the editor has indicated otherwise.

Response: Confirmed.

2. Please review your reference list to ensure that it is complete and correct. If you have cited papers that have been retracted, please include the rationale for doing so in the manuscript text, or remove these references and replace them with relevant current references. Any changes to the reference list should be mentioned in the rebuttal letter that accompanies your revised manuscript. If you need to cite a retracted article, indicate the article's retracted status in the References list and also include a citation and full reference for the retraction notice.

Response: Done.

Responses to comments from Reviewer 3

1) No correction for multiple testing (e.g., FDR or Bonferroni) in feature importance p-values increases risk of false positives among the 25 features.

Response: We thank the reviewer for this important comment regarding multiplicity control. Our primary objective in this study is to identify clinically relevant variables associated with postoperative infection risk under potentially complex and nonlinear relationships, rather than to conduct confirmatory hypothesis testing for a fixed set of prespecified predictors. The permutation feature importance test (PermFIT) framework used in our analysis evaluates the contribution of each feature to the predictive function estimated by the machine learning model, enabling detection of variables that meaningfully affect prediction performance under complex associations.

Because the analysis focuses on variable discovery and interpretability of a predictive model, we followed a commonly used practice in machine learning based feature importance studies by reporting the nominal significance level ($\alpha = 0.05$) for each feature. This approach allows potentially relevant predictors to be identified without overly conservative filtering that could mask meaningful signals, especially when predictors may be correlated or interact nonlinearly within the predictive model.

Importantly, several of the features identified as significant by the adopted PermFITDNN framework including preoperative infection, age, hypertension, obesity, and congestive heart failure have also been reported in prior clinical studies as important risk factors for postoperative infection. The consistency between our findings and previous clinical studies provides external support for the relevance of the identified signals.

Nevertheless, we agree that addressing multiplicity is valuable for readers. Therefore, we have added clarification in the revised manuscript noting that: **The feature importance analysis should be interpreted primarily as an exploratory variable identification procedure within a predictive modeling framework, rather than strict confirmatory hypothesis testing.** We have added a short statement in the revised manuscript to clarify this point and explicitly acknowledge the exploratory nature of the feature importance inference.

2) Marginal performance gains of DNN over XGBoost (e.g., AUC 0.799 vs. 0.787) do not strongly justify its superiority, especially given computational complexity.

Response: Thanks for this thoughtful comment. We agree that the predictive performance of the DNN and XGBoost models is relatively similar in our study, with mean AUC values of 0.799 and 0.787, respectively. However, the primary goal of our work is not solely to achieve marginal improvements in prediction accuracy, but rather to *identify clinically relevant factors associated with postoperative infection risk under potentially complex and nonlinear relationships*. In this context, the advantage of the proposed DNN framework becomes more evident when considering its ability to identify meaningful features through the PermFIT feature importance testing procedure.

Although XGBoost achieved prediction performance close to that of the DNN model, the PermFITDNN framework consistently identified several clinically validated factors that were not detected by XGBoost. For example, the DNN model identified depression and cancer as significant factors associated with postoperative infection risk across the cross-validation folds, whereas the XGBoost model failed to detect these variables as important. Importantly, both depression and cancer have been reported in previous clinical studies as risk factors associated with postoperative infections, suggesting that the DNN-based framework is better able to capture clinically meaningful signals embedded in complex relationships among predictors.

This difference is particularly relevant because the proposed framework aims to provide *interpretable insights into infection mechanisms* in addition to predictive modeling. In clinical applications, identifying meaningful risk factors is often as important as, or even more important than, marginal improvements in predictive accuracy, since these insights can inform clinical decision-making and targeted interventions.

Furthermore, the computational burden of the DNN model in our study is manageable in practice because the model is trained on a moderate-sized dataset with a limited number of predictors ($p = 25$), and the stable DNN ensemble procedure can be implemented efficiently using modern computing environments. Therefore, the modest additional computational cost is justified by the improved capability of the DNN-based framework to identify clinically relevant factors associated with postoperative infection risk.

To clarify this point for readers, we have revised the Discussion section to emphasize that the primary advantage of the PermFITDNN framework lies in its improved ability to identify clinically meaningful predictors under complex association structures, rather than solely achieving marginal gains in prediction accuracy.

3) Some references are worth mentioning: DOI: 10.62762/TETAI.2024.532253; 10.62762/BISH.2025.457428; DOI: 10.62762/BISH.2025.352565

Response: We thank the reviewer for bringing these references to our attention. Our primary scientific interest in this study is to identify clinically meaningful factors associated with postoperative infection risk under potentially complex and nonlinear relationships among predictors. In particular, our proposed framework combines flexible machine learning models with the PermFIT feature importance testing procedure to uncover important variables embedded within these complex associations while maintaining interpretability.

4) The reviewer is wondering how about the computational complexity of the proposed method?

Response: Thanks for raising this important question regarding the computational complexity of the proposed method. Compared with other machine learning methods such as XGBoost, DNNs can indeed involve higher computational cost due to the iterative optimization required for training the network parameters. However, in the present study the computational burden is moderate and manageable

in practice for several reasons. First, the dataset analyzed in our study contains a relatively small number of predictors ($p = 25$) and a moderate sample size, which substantially limits the computational cost of training the DNN model. Second, the stable DNN procedure used in this study is based on bootstrap aggregation and filtering of candidate models, and the training of these models can be efficiently parallelized in modern computing environments. In our implementation, the overall training process can be completed within a reasonable time using standard computing resources.

More importantly, the primary goal of our study is not only predictive accuracy but also the identification of clinically meaningful risk factors embedded in potentially complex nonlinear relationships among predictors. In this context, the PermFITDNN framework demonstrated an important advantage: although the predictive performance of DNN and XGBoost was similar, the DNN model consistently identified several clinically verified factors, such as depression and cancer, as important variables associated with postoperative infection risk, whereas the XGBoost model often failed to identify these factors.

Therefore, the modest additional computational cost of the DNN-based framework is justified by its improved ability to detect clinically meaningful predictors under complex association structures.

5) Imbalanced outcome (inferred 25% infection rate) not explicitly addressed; techniques like SMOTE or weighted loss could improve sensitivity.

Response: We thank the reviewer for raising this important point regarding the imbalanced outcome in our dataset (approximately 25% postoperative infection cases). We agree that class imbalance is an important consideration in predictive modeling, and techniques such as SMOTE or weighted loss are often used in machine learning studies to improve classification sensitivity.

However, the primary goal of our study is not only to achieve high predictive accuracy but also to identify clinically meaningful risk factors embedded in potentially complex nonlinear relationships among predictors under real clinical practice settings, where the outcome distribution is naturally imbalanced. In other words, we aim to identify important factors that help explain why a subset of surgical patients (approximately 25%) develop postoperative infections while the majority (approximately 75%) do not, under the true data-generating relationships between predictors and infection risk.

The framework adopted in this study, namely PermFIDNN, allows us to model complex nonlinear associations while providing statistically principled feature importance inference. Importantly, we intentionally preserved the original outcome distribution in the dataset because oversampling-based methods such as SMOTE artificially modify the joint distribution of predictors and outcomes. Such modifications may distort the true association structure between predictors and postoperative infection risk, which could in turn affect the validity of the identified important features.

Instead, we evaluated model performance using metrics that are appropriate for imbalanced outcomes, particularly the precision-recall area under the curve (PR-AUC). In our analysis, the PermFITDNN framework achieved a PR-AUC of 0.655, which is substantially higher than the baseline prevalence rate of postoperative infection (≈ 0.25) that would be expected from a random classifier. This indicates that the

proposed framework is capable of effectively identifying patients at higher risk of postoperative infection while maintaining the interpretability needed for identifying clinically meaningful predictors.

We have added clarification in the revised manuscript to explicitly discuss the outcome imbalance and the rationale for not applying oversampling-based approaches, emphasizing that preserving the natural data structure is important for reliable interpretation of risk factors.
