# Supplementary material for: An interpretable deep learning framework for predictive modeling of postoperative infections in ICU patients
Source: PLoS One. 2026 Apr 9;21(4):e0346896. doi: 10.1371/journal.pone.0346896 (PMC13065004; doi:10.1371/journal.pone.0346896)
Supplement: Attachment — Submitted filename: Plos one revision.docx [file pone.0346896.s001.docx]

As a reviewer evaluating this manuscript titled **“An Interpretable Deep Learning Framework for Predictive Modeling of Postoperative Infections in ICU Patients”**, submitted to *PLOS ONE*, I provide the following **critical scientific assessment** along with **specific questions and suggestions** to strengthen its rigor, clarity, and reproducibility.

This manuscript addresses an important clinical challenge—predicting postoperative infections in ICU patients—by integrating deep learning with a novel permutation-based feature importance test (PermFIT) to improve both prediction and interpretability. The study uses a large, publicly available dataset (MIMIC-III), implements multiple machine learning models, and performs systematic comparisons. The results indicate that the proposed stable DNN outperforms other models in predictive metrics and interprets key risk factors effectively.

The study is well-motivated and relevant to *PLOS ONE*'s scope, but several **critical issues** require clarification or improvement before the manuscript can be considered for publication.

**Major Scientific Comments & Questions**

**1.** While the PermFIT approach is emphasized as novel, the authors cite their own previous work (Mi et al., 2021; Ref [41]) that also introduced a similar DNN model. How does this work advance that prior publication?

**2.**  Despite high AUC and specificity, sensitivity is **very low (~32%)**, meaning a large proportion of infected patients would be missed. Is this acceptable for a clinical prediction model?

**3.** The authors mention 25 input features but do not explain why these were chosen or whether other known infection risk factors (e.g., **surgical duration**, **antibiotic use**) were considered.

**4.** How were categorical variables encoded? Were patients with multiple ICU admissions properly deduplicated? Could leakage from label-informed preprocessing have inflated results?

**5.** The study uses MIMIC-III exclusively. Have the authors tested the model on **MIMIC-IV** or another external dataset?

**6.**  How were *p*-values corrected for multiple hypothesis testing (e.g., Bonferroni or FDR)? With 25 features, there’s a high risk of Type I error.

**7.** The authors report high specificity but low sensitivity. Were additional metrics such as **F1-score**, **Brier score**, or **calibration curves** considered?

**Minor Issues and Suggestions**

- Clarify acronym definitions at first use (e.g., EHR, DNN).
- Move all figures and tables closer to their first mention for better readability.
- Ensure all references (e.g., Ref [39] for MIMIC-III) link to publicly accessible data sources, and include IRB or ethical approval statements if applicable.
- Typo: “healthcare-associated infections” is occasionally misspelled as “healthcare-associate infections.”

**Summary Recommendation**

This study addresses an important topic with promising methodology, but it requires **substantial revisions** before it meets publication standards. The main concerns are:

- **Sensitivity and clinical applicability of the model**
- **Transparency about methodological choices**
- **Clarification of novelty**
- **Lack of external validation or calibration**

If these concerns are properly addressed, the manuscript would be a strong candidate for publication.
